# Endoscopic Spine Surgery on Instagram: Analysis of Content and Engagement

**DOI:** 10.7759/cureus.62253

**Published:** 2024-06-12

**Authors:** Tara Shenker, Augustus J Rush III, Peter B Derman, Alexander M Satin, Mary P Rogers-LaVanne

**Affiliations:** 1 College of Osteopathic Medicine, Nova Southeastern University, Fort Lauderdale-Davie, USA; 2 Department of Spine Surgery, Texas Spine Consultants, Addison, USA; 3 Department of Spine Surgery, Texas Back Institute, Plano, USA; 4 Department of Research, Texas Back Institute, Plano, USA

**Keywords:** health communication, instagram, social media, endoscopic spine surgery, spine surgery

## Abstract

Introduction

Social media platforms have changed the way society communicates and collaborates. Prior research in healthcare discusses how social media can empower patients, dispel health-related misinformation, and help maintain a patient-centered practice. The goal of this study was to evaluate the use of #endoscopicspinesurgery on Instagram and create a blueprint for creating engaging posts on the social media platform.

Methodology

Public Instagram posts (*n *= 171) that utilized #endoscopicspinesurgery were collected over three months in 2022. Each post was assessed for photo and caption content, likes, comments, number of followers, and hashtag information. Engagement rates were calculated for each post to assess the active interaction of post characteristics and content.

Results

The majority of posts were published by medical professionals (72/171, 42.1%) and industry-related user accounts (55/171, 32.2%). Content related to training, conferences, and the operating room garnered the highest average engagement. Post characteristics (number of hashtags and number of post photos) were significantly associated with engagement.

Conclusions

Results highlighted general trends in creating engaging social media posts, such as using hashtags intentionally to increase searchability and visibility, having higher numbers of photos in a post and using high-quality photos, and understanding the dynamic social media algorithms that may affect post viewership. When structuring social media posts, users should be aware of the audience they want to attract and construct their content accordingly.

## Introduction

Social media platforms, including sites like Instagram, Facebook, X (Twitter), and TikTok, have changed the way society communicates and collaborates. There are pros and cons of using social media in medicine, with potential benefits in patient and physician education, the encouragement of active patient participation, expanding patient care and communication, creating patient support groups, dispelling health-related misinformation, empowering patients, and helping to maintain a patient-centered practice [1-3]. Concerns include risks to patient privacy, debate on the blurring of professional boundaries, questions regarding the quality of information on social media platforms, and potential conflicts of interest in promotional posts [1,4-6].

Instagram is a very popular social media site: the number of Instagram users reached 1.25 billion in 2022, with a predicted growth to 1.57 billion users by 2027 [7]. Within spine surgery, studies have evaluated Instagram posts related to surgeries like spinal fusion and microdiscectomy [8-11]. Some patients use Instagram to share their surgical recovery experiences, and social media posts can be used to identify outcomes important to patients, as well as patients’ general perception of surgery. Individual practices may also benefit from a social media presence. Spine surgeons on social media garnered higher numbers of patient online reviews [12]. There was a positive association between social media and patient ratings for surgeons practicing at academic neurosurgery departments [13], as well as between spine surgeon’s Instagram presence and online physician ratings [14].

The purpose of this study was to identify trends in #endoscopicspinesurgery on Instagram as an example within the field of orthopedics and spine. The results will provide a data-driven guide for spine surgeons on how to optimize their social media usage and connect with their target audience.

## Materials and methods

Data

Public Instagram posts tagged with #endoscopicspinesurgery and published between January 1 and March 31, 2022, were identified using the service Picodash [15]. Many social media platforms include the use of hashtags to increase searchability. A hashtag, the pound symbol followed by a short word or phrase, is added to post captions on Instagram to categorize content and make it more accessible to potential audiences. These hashtags allow users to target specific groups of people by allowing public content to be displayed in searches for a specific hashtag. A total of 171 posts created by 62 unique Instagram accounts were published in the English language and reviewed for the study.

Instagram posts and associated user accounts were evaluated in multiple ways. Each user account was assessed for the number of followers, number of users they follow, type of account (e.g., industry, medical professional), type of medical professional (e.g., spine surgeon, neurosurgeon, pain management), and number of posts in the given study period. Each post was assessed for photo content, caption content, number and wording of hashtags, number of photos, number of likes, and number of comments.

Posts and caption content were classified using deductive content analysis with categories designed a priori based on a preliminary review of Instagram posts from 2021. Content categories for photos were as follows: intraoperative photos, operating room (OR) photos, OR videos, photos of incisions, magnetic resonance imaging (MRI), X-rays, postoperative photos of a patient, postoperative videos of a patient, photos of removed discs, patient-directed advertisements, physician-directed advertisements, cadaver lab training photos, physician profiles, photo of participants at training course/lecture/conference, and unrelated to spine surgery. Content categories for captions were as follows: history and presentation of the patient, discussion of incision size, discussion of surgery time, discussion of recovery time, comparison of endoscopic vs conventional spine surgery, patient-directed advertisements, physician-directed advertisements, discussion about the upcoming training course, discussion of completed training course, unrelated to spine surgery, and no caption.

Statistical analyses

Post engagement was measured with engagement rate, which provides a measure of active interactions with the post controlling for audience reach. Engagement rate = [(number of likes + number of comments)/(number of followers)]*100.

Industry and medical professionals’ posts were assessed for differences in engagement, followers, number of hashtags, and post frequency. In descriptive tables, the mean of each user account’s posts was calculated to contend with the similarities between posts from the same user account. To test for differences between each variable, Poisson generalized linear mixed-effects models (GLMM) were run with the type of account (industry versus medical professionals) as the independent variable. User account was included as a random variable to address the dependency among observations in each unique user account’s posts.

Variables associated with post-engagement in medical professional accounts were investigated using a Gamma GLMM with the user account included as a random variable. Clusters of content types were identified using Jaccard similarity for binary data, grouping variables commonly cooccurring in posts such as both comments and photos with patient-directed advertisements or X-rays with captions containing surgical time (Appendix A). Variables evaluated for inclusion in the final models were: the content clusters, number of followers, number of photos in the post, total number of hashtags used in the post, and number of posts in the study timeframe. Akaike Information Criterion (AIC) and Bayesian Information Criterion (BIC) were obtained for model comparisons with the final selection prioritizing lower AIC and BIC.

A word cloud was created to visualize hashtags often used with #endoscopicspinesurgery. Hashtags were ranked by average engagement rate to uncover the best-performing hashtags. Hashtags for locations, clinic or hospital names, and surgeon names were respectively replaced by the terms #location, #clinic, and #surgeonname to better highlight common trends. For the same reason, all special characters were removed, and all uppercase characters were transformed into lowercase characters. Hashtags used a minimum of four times were included to decrease the likelihood that the hashtags were only used by one account. A list of hashtags posted alongside #endoscopicspinesurgery is located in Appendix B.

An alpha of 0.05 was used to designate significance. Analyses were conducted in R version 4.1.1 [16]. Packages used included dplyr, flexplot, ggplot2, lme4, and wordcloud2 [17-21].

## Results

Description of study variables

A total of 171 posts created by 62 unique Instagram accounts were reviewed (Table 1). Instagram accounts of medical professionals (39/62 users) published 42.1% of all reviewed posts (72/171 posts), while industry-related user accounts (8/62 users) published 32.2% of all reviewed posts (55/171 posts). Comparisons between posts by medical professionals and the industry reveal that industry accounts publish significantly more frequently (*P *= 0.006, Appendix C).

**Table 1:** Distribution of the number of users and the number of posts for the type of account, the type of medical professional, and the location of the user. The majority of posts were published by users located in Asia, North America, and Europe. N/A, not applicable

Variable	Users (*n*)	Users (%)	Posts (*n*)	Posts (%)
Type of account	62		171	
Industry (medical device)	8	12.90%	55	32.20%
Medical professional	39	62.90%	72	42.11%
Other (e.g., advertising companies, hospital accounts)	15	24.19%	44	25.73%
If medical professional, what type:	39		72	
Spine/Orthopedic surgeon	23	58.97%	45	62.50%
Neurosurgeon	10	25.64%	20	27.80%
Pain management	4	10.26%	4	5.60%
Other medical professionals	2	5.13%	3	4.20%
Location	62		171	
Africa	2	3.23%	8	4.70%
Asia	32	51.61%	68	39.80%
Australia	2	3.23%	5	2.90%
Europe	5	8.06%	24	14.00%
North America	17	27.42%	62	36.30%
South America	2	3.23%	2	1.20%
N/A (advertising companies)	2	3.23%	2	1.20%

Medical professionals published an average of 1.85 (standard deviation [SD] 1.76) posts per user with a range of 1-8 posts (Table 2). The majority (87.5%) of posts by medical professionals with public commenters garnered at least one comment from another medical professional, including people who defined themselves in their Instagram biographies as physicians, spine surgeons, surgeons, and physical therapists.

**Table 2:** Descriptive variables for posts by medical professionals. SD, standard deviation

Variable	Mean	SD	Range
Engagement score	9.71	11.71	0.53-69.44
Number of comments	1.47	2.37	0-14
Number of likes	35.84	53.39	1-297
Number of followers	616.59	767.98	21-3,800
Number of accounts following	317.76	556.95	0-3,071
Number of photos in the post	2.16	2.04	1-10
Number of hashtags in the post	12.40	7.97	0-30
Number of posts in the study timeframe	1.85	1.76	1-8

For medical professionals’ posts, the most common photo content was patient-directed advertisements, followed by MRI images, OR photos, photos unrelated to spine surgery, intraoperative (endoscopic camera) photos, videos in the OR, and photos of a removed disc. The most common caption content was patient-directed advertisements, followed by a history and presentation of patients, discussions of recovery time, and a discussion of incision size (Appendix D).

Medical Professionals’ Post Content Ranked by Engagement

**Figure 1:**
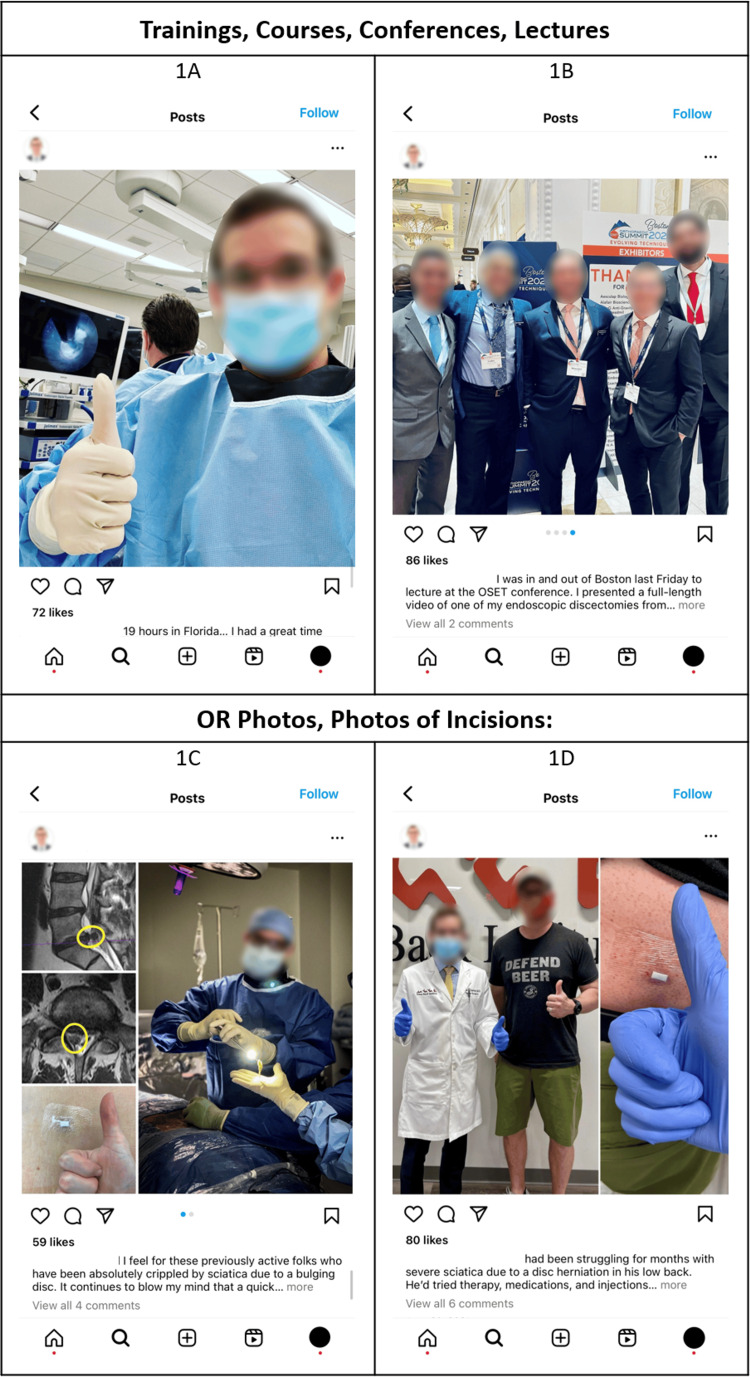
Example of Instagram posts that contain elements with high engagement. High-performing content in this study included (A) training courses/lectures, (B) conferences, (C) operating room (OR) photos, and (D) photos of incisions. Permission for use from the original author of the Instagram posts was obtained [22-25]. Faces and names have been blurred or obscured.

Medical professionals’ posts garnered an average of 1.47 comments and 35.84 likes. The mean engagement rate for posts by medical professionals was 9.71. Content with a high average engagement rate included captions containing discussion about upcoming training course(s), cadaver lab training photos, photos of participants at a training course(s)/lecture(s)/conference(s), captions containing discussion of completed training course(s), and OR photos (Appendix E). To illustrate well-performing content, Figure 1 shows example Instagram posts containing top-ranking content.

Content with low-average engagement rate included X-rays, no caption, caption discussing time of surgery, postoperative patient photos, and postoperative patient videos (Appendix E). Some of the most common content types received the lowest average engagement rate. For example, photos with patient-directed advertisements were quite common, but had low-average engagement.

Variables Associated With Engagement in Medical Professionals’ Posts

The final model-predicting engagement rate included number of followers, number of photos in the post, and total number of hashtags (Table 3). Number of photos (*P *= 0.018) was positively associated with engagement rate. Number of followers (*P *= 0.003) and number of hashtags (*P *= 0.041) were negatively associated with engagement rate.

**Table 3:** The parameters of the generalized linear mixed-effects model for engagement rate given unique user account. Number of followers, number of photos in the post, and number of hashtags were significantly associated with engagement rate. Number of photos was positively associated with engagement rate, and number of followers and number of hashtags were negatively associated with engagement rate. The user account explained a large amount of the variance. ^*^P < 0.05 ^**^P < 0.01. ^***^P < 0.001. β, regression beta coefficient; SE, standard error

	Estimate (β)	SE	*t*-value	*P*-value
Intercept	2.05	0.232	8.820	<0.001***
Number of followers	-0.001	0.000	-3.023	0.003**
Number of photos in the post	0.138	0.058	2.377	0.018*
Number of hashtags	-0.019	0.009	-2.040	0.041*

Common Hashtags Used With #endoscopicspinesurgery by Medical Professionals

**Figure 2:**
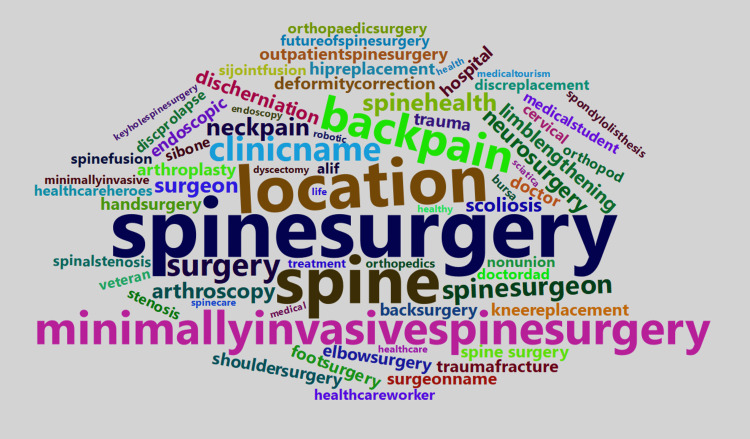
Word cloud displaying common hashtags used with #endoscopicspinesurgery. The size of the words corresponds to the frequency of use. The most commonly co-occurring hashtags (#) in order of frequency were #spinesurgery, #spine, #location (specific in the post to town, city, country), #backpain, #minimallyinvasivespinesurgery, #clinicname (specific in the post to hospital or clinic name), #surgery, #spinesurgeon, #neckpain, and #spinehealth.

Hashtags used most commonly with #endoscopicspinesurgery in order of frequency were #spinesurgery, #spine, location hashtags, #backpain, #minimallyinvasivespinesurgery, and the clinic or hospital name (Figure 2). Hashtags with high engagement included the clinic or hospital name, #endoscopic, location hashtags, and #minimallyinvasive (Table 4).

**Table 4:** List of the top 30 hashtags ranked by average engagement rate. Only hashtags with multiple posts (≥4) were included in this list to reduce bias of individual user accounts who may use the same hashtags on each post. Instagram allows users to include up to 30 hashtags on a post. #, hashtag

Hashtag	Average engagement rate	Range	*n* (posts)
Surgeon name as a hashtag	19.30	0.53-69.44	11
#dyscectomy	14.00	8.00-16.00	4
#health	14.00	8.00-16.00	4
#healthcare	14.00	8.00-16.00	4
#healthy	14.00	8.00-16.00	4
#life	14.00	8.00-16.00	4
#medical	14.00	8.00-16.00	4
#medicaltourism	14.00	8.00-16.00	4
#robotic	14.00	8.00-16.00	4
#orthopedics	13.14	8.00-16.00	5
#neurosurgery	12.61	1.40-69.44	9
#bursa	11.59	1.97-16.00	5
#treatment	11.31	0.53-16.00	5
#minimallyinvasive	10.61	3.53-18.02	5
#minimallyinvasivespinesurgery	10.36	2.24-69.44	19
#spinesurgery	9.73	1.64-69.44	37
#surgery	8.63	0.53-16.00	13
#surgeon	7.91	1.98-16.00	9
Location	7.87	0.67-69.44	86
#hospital	7.81	1.00-16.00	8
#outpatientspinesurgery	7.72	5.21-14.73	7
#spinehealth	6.83	0.67-18.46	10
Clinic/hospital name	6.82	1.00-27.91	18
#futureofspinesurgery	6.56	5.21-8.40	6
#spine	6.49	0.53-18.46	27
#sciatica	6.40	2.24-16.45	4
#endoscopic	6.15	2.49-14.29	7
#discprolapse	5.66	2.63-9.72	7
#spinecare	5.60	1.64-13.18	4
#backpain	5.53	1.31-18.02	19

## Discussion

In this study, #endoscopicspinesurgery was used as an example within the field of spine and orthopedics to identify trends in high-performing social media content on the Instagram platform. A data-driven guide to Instagram for spine surgeons is provided based on these results, specifically focusing on (1) creating content for a targeted audience, (2) using hashtags effectively, and (3) understanding how post characteristics affect engagement rate.

#endoscopicspinesurgery targets spine surgeons

Medical professionals in this study had a 9.71 overall mean Instagram engagement rate. This is fairly high - comparatively, influencers with 1-10,000 followers averaged a 2.53 engagement rate in 2022 [26]. One of the keys to success on social media is deciding upon and curating posts for a targeted audience. In this study, we found that #endoscopicspinesurgery is a way for medical professionals to communicate and collaborate in a public setting, and industry accounts used #endoscopicspinesurgery to reach physicians on Instagram. While previous studies have reviewed the potential benefits and risks of interactions between physicians and patients on social media [3], no patients posted with #endoscopicspinesurgery in the given study timeframe. Content related to training and conferences and OR photos garnered high average engagement rates, reflecting how this type of content may be applicable and appealing to other professionals within the field of spine surgery.

Despite these findings, the highest-represented content in both photos and comments was patient-directed advertisements. These, particularly photo advertisements, were among the lowest engagement rates. Although #endoscopicspinesurgey represents the type of surgery being advertised or discussed, patients may be more likely to use generalizable terms like *spine surgery* or *fusion*. Further, photos containing text tend to perform worse on Instagram [27].

The high amount of patient-directed advertisements identified in this study suggests that medical professionals using Instagram want to target potential patients but are effectively targeting other physicians. For a general audience, previous studies in other medical fields have identified engaging content such as patient and physician *selfies*, physicians in scrubs and white coats, short videos, short captions, and captions with personal experiences [28]. Physicians can evaluate their prior posts to assess if they are reaching their target audience and adjust accordingly with improved hashtag use, increased number and quality of photos in each post, and content that garnered higher engagement like training and conference posts, OR photos, photos of small incision sizes, and photos of removed discs.

Hashtag use and post-engagement

Hashtags increase the searchability and visibility of posts, but users must be selective in the hashtags they choose. In this study, there was a negative association between number of hashtags and engagement rate. The most prevalent number of hashtags used on physician accounts were four (10/72 posts), 13 (7/72 posts), and 30 (7/72 posts) hashtags. Perhaps those using many hashtags are new to Instagram and trying to break into the space - three of the seven posts with 30 hashtags had ≤25 followers. Using highly popular hashtags might cause the content to be buried in millions of posts, while a small number of hashtags might cause the content to be too niche and reach a smaller audience [29]. Instagram allows up to 30 hashtags per post, and a study recommended 20 hashtags to optimize engagement, although post quality mattered more than hashtags [29].

In the posts evaluated, #spinesurgery was used most frequently alongside #endoscopicspinesurgery, followed by location hashtags and #spine. These hashtags were also in the top 30 hashtags for engagement rate. #spinesurgery and #spine have over 185,000 and 1.3 million posts on Instagram, respectively. These tags are used widely across many disciplines of medicine and incorporate a large variety of post content. Comparatively, #endosopicspinesurgery has about 5,000 posts associated with the hashtag.

One of the reasons for the massive increase in popularity of #spinesurgery and #spine is the generalizability. These tags can be associated with a larger group of content and are less specific than #endoscopicspinesurgery. Previous studies show that hashtags that use lay persons terminology are associated with a higher number of posts than hashtags including proper medical terminology [30]. Users can potentially increase visibility by incorporating generalizable and commonly searched hashtags.

Understanding other factors influencing engagement

Several followers and the number of photos in the post were also identified as variables associated with engagement. Engagement was higher for posts with greater numbers of photos. There are multiple possible explanations for this finding. First, users spend increased time looking at a post with multiple photos and may therefore feel more inclined to like and comment compared with seeing one photo and continuing to scroll. Further, in another analysis of Instagram posts outside of the medical field, post characteristics (e.g., quality of the photos, text on the photo) were identified as potentially more important for engagement rate than specific content categories [27], matching our finding of the impact of multiple photos.

Several followers were negatively associated with engagement rate, which may be due to a higher inactive following for accounts with a large following: to have a high engagement rate, a larger percentage of an account’s followers need to like and/or comment on a post. If the user’s followers are inactive, unengaged, or sourced from a for-profit account following the service, they will not like or comment on a post. A low engagement rate can impact viewers’ impression of the Instagram account’s credibility [31].

A low engagement rate can also be due to factors associated with the individual's account, such as the user’s past engagement, the user’s activity, and the type of accounts the user follows. Like many social media sites, Instagram uses proprietary and dynamic algorithms, classifiers, and processes to display ordered, specialized content for users [32]. The Instagram algorithms likely include more than likes and comments when computing which content to display on which page, and factors such as engagement with similar content, post timing, prior content engagement rates, and profile interactions have all been identified as variables that may influence post visibility and engagement [32,33].

Platform activity may also impact engagement. One suggested way to increase visibility is to follow accounts that commonly interact with content similar to your posts. Instagram has an *Explore* page that includes posts from users you may not follow, but may be interested in. Interacting with similar content may increase the likelihood of your content appearing on others’ explore pages, thus increasing visibility and hopefully future engagement [32]. While number of posts per month was not associated with differences in engagement in this study, other publications have identified the importance of platform activity [34,35]. A recent cross-sectional study reported that many physicians had a social media profile, but did not post every month [36]. An analysis of neurosurgeon influencers on Instagram revealed that the top 30 influencers post significantly more frequently compared with other neurosurgeons [37].

Other recommendations for surgeons using Instagram

There are additional ways medical professionals can utilize social media strategies, and below we discuss considerations beyond trends identified in #endoscopicspinesurgery. First, social media can be an effective way to communicate with a large and diverse audience. A recent study critiqued representation in medical Instagram posts; when curating a social media presence, mindfully considering if posts reflect the patient demographic is important [38]. A recent study warned that bias in demographics represented in social media posts can cause misjudgments of viewers’ health risks [39].

In 2016, social media platforms updated to include the use of *stories*, which allow users to post photos or videos that disappear based on a user-decided timeframe of up to 24 hours. Using polls, asking questions, and live communication are other ways physicians can promote audience interaction on Instagram. This strategy can also be used in post captions: asking the audience questions in the caption can generate more comments and increase engagement rate and possible audience reach.

Finally, physicians who have their Instagram set up as *content creator* can currently view the analytics of their posts. This information can help spine surgeons evaluate what generates engaging content for their personal accounts.

Limitations

This study is limited to public posts and is missing data on private, removed, and time-limited posts (e.g., stories). There is a limited sample size and study timeframe, and this study included only posts in the English language. While a global sample was included, there were under- and over-representations in continental locations. This manuscript focuses solely on variation in engagement related to content, while other studies have pointed to variation in engagement due to creator-related features such as the poster’s age or gender [33,38]. Finally, Instagram’s algorithms, classifiers, and processes may change over time, so the identified trends may also change over time.

## Conclusions

Instagram and other social media platforms have become an integral way that society communicates and interacts with each other. These evolving platforms have also become an important way that medical professionals can interact with each other and patients. This study evaluated the use of #endoscopicspinesurgery on Instagram to analyze current trends and design a generalizable guide for spine surgeons seeking to improve their use of the Instagram platform. These results highlight hashtag choice, target audience, and post content and characteristics as critical aspects of engaging social media content.

## Figures and Tables

**Figure 3:**
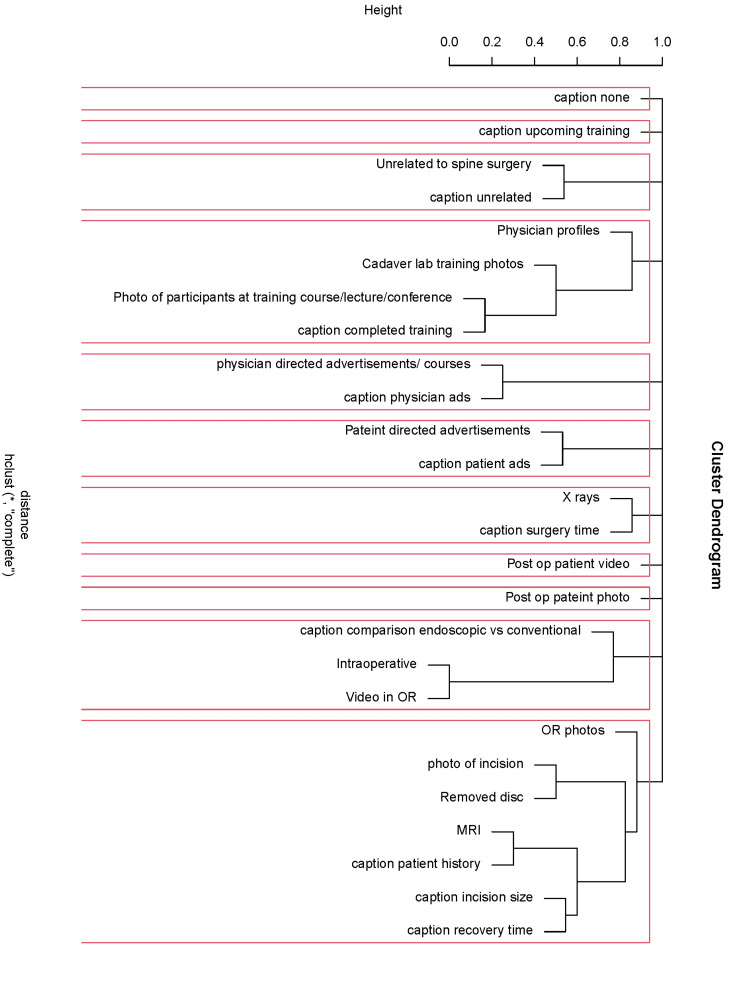
Dendrogram identifying clusters of commonly co-occurring photo and caption content categories.

**Figure 4:**
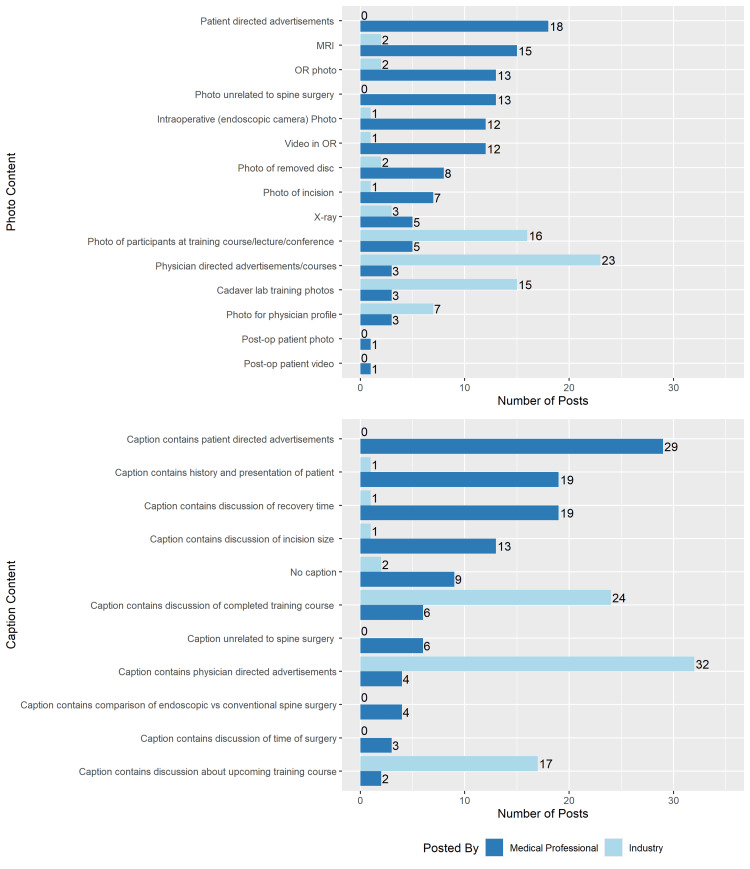
The count of each photo and caption content in posts published by medical professionals and industry accounts. The most represented content in medical professionals’ posts was patient-directed advertisements, while the most represented content for industry account posts was physician-directed advertisements.

**Figure 5:**
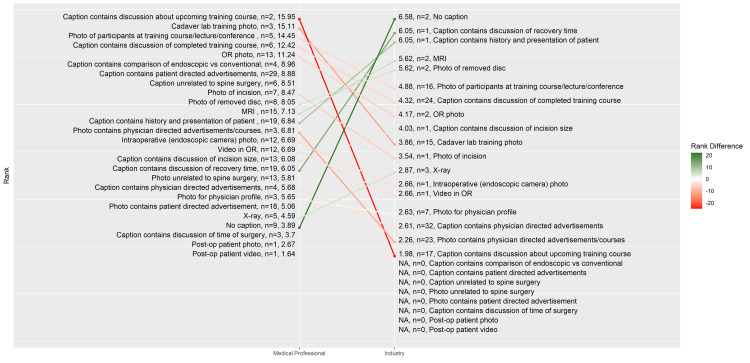
Comparison between medical professionals (left) and industry (right) posts of average engagement rates for each type of photo and caption content. Photo and caption contents are displayed and ranked from most to least engagement. A number of contents were not represented in any industry posts. The scaled differences between the rankings are highlighted where increases in engagement rate rank are indicated in green and decreases in engagement rate rank are indicated in red. The largest difference is that captions containing discussion about upcoming training course received much higher engagement for medical professional’s posts. The sample size for each content is noted.

## Appendices

Appendix A 

Appendix B 

**Table 5:** List of hashtags (#) used with #endoscopicspinesurgery in posts by medical professionals and listed in order of frequency.

Count	Hashtag
34	#spinesurgery
27	#location, #spine
19	#backpain, #minimallyinvasivespinesurgery
14	#clinicname
13	#surgery
11	#spinesurgeon
10	#neckpain, #spinehealth
9	#arthroscopy, #neurosurgery, #surgeon
8	#discherniation, #limblengthening, #scoliosis
7	#arthroplasty, #backsurgery, #deformitycorrection, #doctor, #elbowsurgery, #endoscopic, #footsurgery, #handsurgery, #hipreplacement, #hospital, #kneereplacement, #outpatientspinesurgery, #shouldersurgery, #spine surgery, #surgeonname, #trauma, #traumafracture
6	#alif, #cervical, #discprolapse, #discreplacement, #doctordad, #futureofspinesurgery, #healthcareheroes, #healthcareworker, #medicalstudent, #nonunion, #orthopaedicsurgery, #orthopod, #sibone, #sijointfusion, #spinalstenosis, #spinefusion, #stenosis, #veteran
5	#bursa, #minimallyinvasive, #orthopedics, #spondylolisthesis, #treatment
4	#dyscectomy, #endoscopy, #health, #healthcare, #healthy, #keyholespinesurgery, #life, #medical, #medicaltourism, #robotic, #roboticsurgeryturkey, #sciatica, #spinecare
3	#awakespinesurgery, #hernia, #herniateddis, #laserdisc, #lumbar, #minimallyinvasivesurgery, #narcoticfreespinesurgery, #neurosurgeon, #operatingroom, #pain, #painmanagement, #patientcare, #physiotherapy, #pseudoarthrosis, #radiculopathy, #slipdisc, #spinalexercises, #spinalhealth, #spinalproblems, #spinalsurgery, #team
2	#backhealth, #backpainrelief, #besthospitality, #bestsurgeon, #bronchoscopy, #cadaver, #chronicpain, #conference, #dentalclinic, #dentistry, #dietitian, #disc, #endoscope, #endoscopicspineexperts, #endoscopicsurgery, #epilepsy, #fillersinjection, #fluoro, #herniateddisc, #hospitality, #implants, #joimax, #jointreplacementsurgeon, #keyholesurgery, #knee, #legpain, #medicalconference, #microscopicspinesurgery, #neurologist, #nonarcotics, #or, #painlessdelivery, #physiotherapist, #psld, #regenerativemedicine, #safespinesurgery, #slippeddisc, #spinal, #spinalphysiotherapy, #spineendoscopy, #surgeonlife, #surrogacy, #teamcare, #wprc2022, #yoga
1	#artificialdiscreplacement, #back, #backsareourbusiness, #besthospital, #bestneurologist, #bestneurosurgeon, #bestorthohospital, #bestorthopaedicshospitalpunjab, #bestorthopaedicsurgeon, #bestspinesurgeon, #boostfertility, #brainandspinedocter, #centreofexcellence, #chroniclowbackpain, #closedherniateddiscsurgery, #developmentalhipdislocation, #discectomy, #discfx, #discsurgery, #doctors, #elliquence, #endoscopicinterlaminarspinesurgery, #endoscopicspine, #epilepticfits, #epilepticseizures, #ess, #extensions, #fertilitypower, #fits, #fitspine, #fitterspine, #gettingbetterafterspinesurgery, #gynaec, #gynaecologist, #headache, #healthyliving, #height, #heightextension, #hip, #hipdislocation, #implantsinspinesurgery, #incisions, #interventionalpainmanagement, #kneepain, #kneereplacment, #kneesurgery, #latesttechnologyforjointreplacement, #lesss, #limb, #lowbackpain, #meded, #medicine, #mentalhealth, #mentalhealthmatters, #microdiscectomy, #microsized, #microsizedincisions, #migranine, #modernmedicine, #mp, #navigatedspinesurgery, #navio, #neckpainrelief, #nephrology, #neurochirurgrunner, #neurology, #newskills, #newtechnology, #nolasershere, #obstetriciangynecologist, #oldagespinalproblems, #oncosurgery, #opthalmologist, #orthography, #orthopadeic, #orthopadeicspecialist, #orthopaedic, #orthopaedics, #orthopaedicsurgeon, #orthopedicsurgery, #painfree, #paralyis, #parkinsons, #patientlove, #patientswhoflourish, #pediatric, #pediatricorthopedics, #photography, #physicaltherapy, #procedure, #prolapsedintervertebraldisc, #prptreatment, #regen, #relieveschroniclowbackpain, #riwospine, #robotickneereplacement, #robotics, #roboticsurgery, #routineactivitiesafterspinalsurgery, #runchangesyourbrain, #safemotherhood, #sciaticarelief, #shoulder, #slippeddiscsurgery, #smalltubularsystems, #smithnephew, #specialist, #spinalcord, #spinalcordinjuryexercises, #spinalfusion, #spinalinjections, #spinalmovement, #spinalpain, #spinalposture, #spinalpostures, #spinalrecovery, #spinalsurgeryrecovery, #spinalwellbeing, #spineadvancements, #spinecenter, #spineexpert, #spinepain, #spineproblem, #spineproblems, #spinerobotics, #spinetreatment, #sportsinjuries, #stresstest, #stroke, #successfulspinesurgery, #surgical, #surgicalfield, #surgicalprocedure, #teamwork, #technology, #testtubebaby, #testtubebabycentre, #tovisualisethesurgicalfield, #tubularsystems, #tulangbelakang, #turanturan, #ube, #ubetraining, #ultraminimallyinvasive, #urology, #usingmicrosizedincisions, #vadodaracity, #wellness, #workshop, #workshop2022, #worldwide

Appendix C

**Table 6:** Descriptive variables for posts by industry accounts and medical professionals. The mean and standard deviation (SD) displayed here are the mean (SD) of unique user account means. The number of posts published in the study timeframe was significantly greater for industry accounts (*P *= 0.006). There were no significant differences between the subsets for any other variables (all *P *> 0.05).

	Subset of data: Industry				Subset of data: Physicians and medical professionals			
Variable	N	Mean	SD	Range	N	Mean	SD	Range
Engagement score	55	4.69	4.64	0.14-15.11	72	9.71	11.71	0.53-69.44
Number of comments	55	0.77	1.35	0-4	72	1.47	2.37	0-14
Number of likes	55	38.28	43.12	1-129	72	35.84	53.39	1-297
Number of followers	55	1294.99	1286.54	67-3767	72	616.59	767.98	21-3800
Number of accounts following	55	766.41	1774.08	7-5143	72	317.76	556.95	0-3071
Number of photos in the post	55	2.17	1.72	1-10	72	2.16	2.04	1-10
Number of hashtags in the post	55	8.86	3.99	3-16	72	12.40	7.97	0-30
Number of posts in study timeframe	55	6.88	7.95	1-19	72	1.85	1.76	1-8

Appendix D 

Appendix E
